# Factors affecting the ORR after neoadjuvant therapy of TP regimen combined with PD-1 inhibitors for esophageal cancer

**DOI:** 10.1038/s41598-023-33038-w

**Published:** 2023-04-13

**Authors:** Rulan Ma, Dawei Yuan, Caijing Mo, Kun Zhu, Chengxue Dang, Yong Zhang, Jianhao Yin, Kang Li

**Affiliations:** 1 Department of Surgical Oncology, The First Affiliated Hospital of Xi’an Jiaotong University, 277 West Yanta Road, Xi’an, 710061 Shaanxi China; 2 Department of General Surgery, Shaanxi Provincial Cancer Hospital Affiliated to Medical College of Xi’an Jiaotong University, 309 West Yanta Road, Xi’an, 710061 Shaanxi China

**Keywords:** Cancer, Oncology

## Abstract

The aim of this study is to evaluate the factors affecting the objective response rate (ORR) after neoadjuvant therapy of taxol plus platinum (TP) regimen combined with programmed cell death protein-1 (PD-1) inhibitors for esophageal cancer, and establish a predictive model for forecasting ORR. According to the inclusion and exclusion criteria, consecutive esophageal cancer patients who were treated in the First Affiliated Hospital of Xi’an Jiaotong University from January 2020 to February 2022 were enrolled in this study as a training cohort, while patients who were treated in the Shaanxi Provincial Cancer Hospital Affiliated to Medical College of Xi’an Jiaotong University from January 2020 to December 2021 were enrolled as a validation cohort. All patients were treated with resectable locally advanced esophageal cancer and received neoadjuvant chemotherapy combined with immunotherapy. The ORR was defined as the sum of complete pathological response, major pathological response and partial pathological response. Logistic regression analysis was performed to determine the factors that might be related to the ORR of the patients after neoadjuvant therapy. The nomogram based on the result of regression analysis was established and verified to predict the ORR. In this study, 42 patients were included as training cohort and 53 patients were included as validation cohort. Chi-square analysis showed that neutrophil, platelet, platelet-to-lymphocytes ratio (PLR), systemic immune-inflammation index (SII), D-dimer and carcinoembryonic antigen (CEA) between ORR group and non-ORR group were significantly different. Logistic regression analysis showed that aspartate aminotransferase (AST), D-dimer and CEA were independent predictors of ORR after neoadjuvant immunotherapy. Finally, a nomogram was established based on AST, D-dimer and CEA. Internal validation and external validation revealed that the nomogram had a good ability to predict ORR after neoadjuvant immunotherapy. In conclusion, AST, D-dimer and CEA were the independent predictors of ORR after neoadjuvant immunotherapy. The nomogram based on these three indicators showed a good predictive ability.

## Introduction

Esophageal cancer is one of the most common causes of tumor-related death in the world, and more than half of the patients with esophageal cancer were diagnosed with locally advanced tumors^1^. In the past few decades, researchers have conducted a series of clinical studies to establish a standard treatment for esophageal cancer^2,3^. CROSS trial confirmed that preoperative chemoradiotherapy could significantly prolong the overall survival of patients, making neoadjuvant chemoradiotherapy as the recommended treatment for locally advanced esophageal cancer^4^. Although this clinical trial confirmed that neoadjuvant chemoradiotherapy had the advantage of improving prognosis, nearly half of the patients still had postoperative recurrence or distant metastasis^4^. This suggests that more systematic and effective treatments are still needed to prevent potential recurrence and metastasis of esophageal cancer.

A series of phase III clinical trials have confirmed that immunotherapy represented by programmed cell death protein-1 (PD-1) inhibitors can be used as first-line treatment for advanced esophageal cancer^5–7^. Subsequently, several clinical trials have evaluated the safety and efficacy of neoadjuvant chemotherapy combined with immunotherapy, and confirmed that immunotherapy could be used as a part of neoadjuvant therapy in patients with locally advanced esophageal cancer^8,9^. 25–56% of esophageal cancer patients acquired to complete pathological response (pCR) after neoadjuvant chemotherapy combined with immunotherapy, while 50–90% patients acquired to objective response rate (ORR), indicating that preoperative chemotherapy combined with immunotherapy is a feasible neoadjuvant therapy for esophageal cancer^9–12^.

It is suggested that pathological response is associated with the prognosis of the patients. Therefore, predicting pathological response rate of the patients who received neoadjuvant therapy is necessary. Several studies have reported the predictive models that can predict pCR and tumor regression grade in patients with esophageal cancer after neoadjuvant immunotherapy^13,14^. However, there is still no simple and feasible method to predict the ORR of patients. Therefore, the aim of this study was to establish an effective model for predicting the ORR of esophageal cancer patients after neoadjuvant TP (platinum + taxol) regimen combine with PD-1 inhibitors (Pembrolizumab, Camrelizumab, Tislelizumab or Sintilimab).

## Methods

### Patients

**Figure 1:**
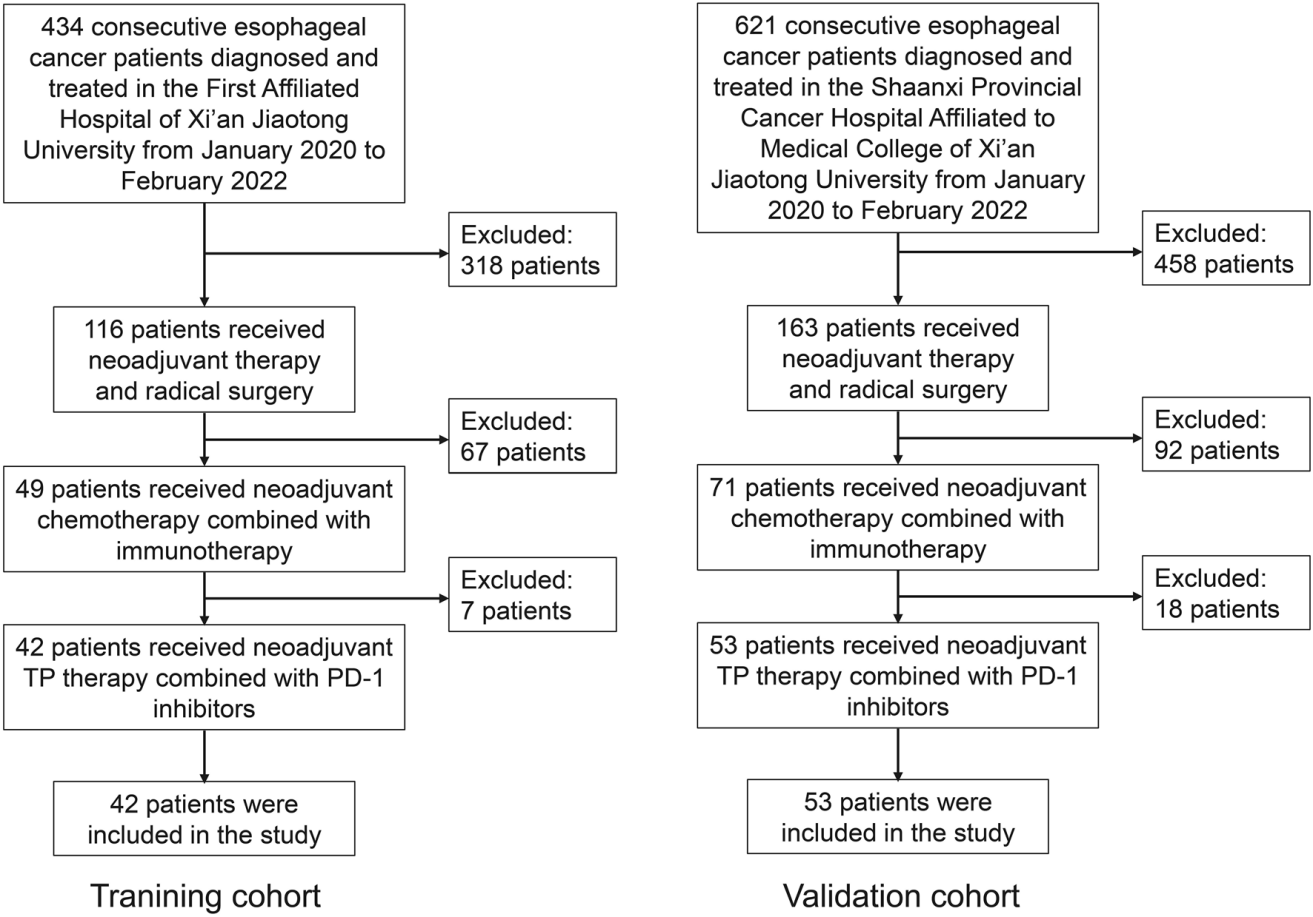
Flow chart of patient selection. TP, taxol + platinum; PD-1, programmed cell death protein-1.

Consecutive esophageal cancer patients who were diagnosed and treated in the First Affiliated Hospital of Xi’an Jiaotong University from January 2020 to February 2022 were enrolled in this study according to the inclusion criteria. The inclusion criteria were as follows: (1) pathological diagnosis: esophageal carcinoma; (2) received neoadjuvant TP regimen combined PD-1 inhibitors; (3) received radical surgery; (4) the clinicopathological and postoperative pathological data were completed; (5) not received any other anti-tumor therapy; (6) no immune system disease. Patients did not meet with the inclusion criteria were excluded in this study. In this study, a total of 42 patients were included as a training cohort. According to the above inclusion and exclusion criteria, we also enrolled 53 consecutive patients treated in the Shaanxi Provincial Cancer Hospital Affiliated to Medical College of Xi’an Jiaotong University from January 2020 to December 2021 as a validation cohort (Fig. 1). We defined the ORR as pCR (no residual tumor cells in the resected specimens and all resected lymph nodes) + major pathological response (≤ 10% viable tumor cells in the resected primary tumor and all resected lymph nodes) + partial pathological response (≤ 50% viable tumor cells in the resected primary tumor and all resected lymph nodes)^15,16^.

### Data collection and processing

The baseline data, clinicopathological data, treatment-related data and laboratory indexes of the enrolled patients were collected. The data was processed by using Microsoft Excel and SPSS 26.0. The optimal cut-off value of continuous data was calculated by receiver operating characteristic (ROC) curve. Then, the continuous data was converted into binary data according to the optimal cut-off value.

### Statistical analysis

The statistical analysis was performed by SPSS 26.0 and Rstudio. The difference between the two groups was analyzed by x^2^ test. Univariate and multivariate logistic regression analyses were conducted to evaluate the factors that might be related to the ORR of the patients after neoadjuvant therapy. According to the result of multivariate logistic regression analysis, a nomogram for predicting ORR was established. The predictive ability was validated by using C-index, ROC curve, calibration curve, decision curve analysis (DCA) and clinical impact curve (CIC). *P*-value less than 0.05 was considered to be statistically significant.

### Ethics approval

This study was performed in accordance with the Declaration of Helsinki, and was conducted under the approval and supervision of the Ethics Committee of the First Affiliated Hospital of Xi’an Jiaotong University (No. XJTU1AF2022LSK-335). The study was a retrospective study, and written informed consent for participation was not required for this study in accordance with the national legislation and the institutional requirements. Therefore, the waiver of informed consent was approved by the Ethics Committee of the First Affiliated Hospital of Xi’an Jiaotong University.

## Results

### Characteristics of the patients with esophageal cancer

A total of 42 patients with esophageal cancer from the First Affiliated Hospital of Xi'an Jiaotong University were enrolled as the training cohort in this study, while 53 patients from Shaanxi Provincial Cancer Hospital Affiliated to Medical College of Xi'an Jiaotong University were enrolled as the validation cohort (Fig. 1). All the patients received neoadjuvant TP regimen combined with PD-1 inhibitors and radical surgery. The mean age of the patients in training cohort was 62.33 ± 7.39 years, and the mean age of the patients in training cohort was 63.56 ± 5.85 years. Postoperative pathological result showed that 54.76% (23/42) and 49.06% (26/53) patients acquired ORR after neoadjuvant therapy in training cohort and validation cohort, respectively. The clinicopathological features and laboratory findings of these patients before neoadjuvant therapy are shown in Tables 1 and 2. Notably, it was found that neutrophil, platelet, platelet-to-lymphocytes ratio (PLR), systemic immune-inflammation index (SII) [SII = (platelet × neutrophil) / lymphocytes], D-dimer and carcinoembryonic antigen (CEA) were significantly different among ORR group and Non-ORR group (Table 2).

**Table 1:** Characteristics of esophageal cancer patients received neoadjuvant chemotherapy combined with immunotherapy.

Term		Training cohort			Validation cohort		
		Non-ORR	ORR	*P*-value	Non-ORR	ORR	*P*-value
Gender	Male	14	21	0.214^a^	20	23	0.181
	Female	5	2		7	3	
Age (Years)	< 52	4	1	0.158^a^	8	2	0.076^a^
	≥ 52	15	22		19	24	
BMI	< 22.57	12	11	0.320	18	12	0.132
	≥ 22.57	7	12		9	14	
Smoking history	No	11	12	0.711	15	13	0.685
	Yes	8	11		12	13	
Drinking history	No	13	18	0.504^a^	18	21	0.244
	Yes	6	5		9	5	
Family history of cancer	No	18	21	1.000^a^	25	23	0.607^a^
	Yes	1	2		2	3	
Tumor location	Upper	3	3	0.475^a^	4	3	0.523^a^
	Median	5	9		7	10	
	Lower	6	9		9	10	
	Middle-lower	5	2		7	3	
Gross type	Medullary	12	10	0.204	17	10	0.074
	Other	7	13		10	16	
Pathological type	Squamous carcinoma	18	23	0.452^a^	26	26	1.000^a^
	Adenocarcinoma	1	0		1	0	
Grade	1	5	14	1.000^a^	6	3	0.485
	2/3	4	15		21	18	
cT stage	1/2	2	3	1.000^a^	3	3	0.967
	3/4	17	20		24	23	
cN stage	0/1	9	8	0.625	13	14	0.571
	2/3	11	7		14	11	
cM stage	0	18	19	0.356^a^	25	21	0.250^a^
	1	1	4		2	5	
cTNM stage	I/II	4	15	1.000^a^	6	5	
	III/IV	4	19		21	21	
TP + PD-1 inhibitors	TP + Camrelizumab	10	11	1.000^a^	15	11	0.854^a^
	TP + Sintilimab	3	4		4	5	
	TP + Tireilizumab	4	5		5	6	
	TP + Pembrolizumab	2	3		3	4	
Neoadjuvant therapy cycle	1–2	11	17	0.273	16	19	0.288
	3–4	8	6		11	7	

ORR, objective response rate; BMI, body mass index; PD-1, programmed cell death protein-1; TP, taxol + platinum.

**Table 2:** Laboratory indicators of esophageal cancer patients received neoadjuvant chemotherapy combined with immunotherapy.

Term		Training cohort			Validation cohort		
		Non-ORR	ORR	*P*-value	Non-ORR	ORR	*P*-value
WBC (× 10^9^/L)	< 6.96	9	17	0.078	13	20	**0.031**
	≥ 6.96	10	6		14	6	
Lymphocyte (× 10^9^/L)	< 1.64	14	14	0.381	18	14	0.340
	≥ 1.64	5	9		9	12	
Neutrophil (× 10^9^/L)	< 5.22	10	20	**0.014**	14	22	**0.011**
	≥ 5.22	9	3		13	4	
Hemoglobin (g/L)	< 137.00	7	12	0.320	9	14	0.132
	≥ 137.00	12	11		18	12	
Platelet (× 10^9^/L)	< 237.50	7	16	**0.034**	10	19	**0.008**
	≥ 237.50	12	7		17	7	
NLR	< 2.42	7	15	0.067	10	16	0.074
	≥ 2.42	12	8		17	10	
PLR	< 166.50	8	17	**0.037**	12	20	**0.016**
	≥ 166.50	11	6		15	6	
SII	< 477.40	4	12	**0.039**	6	13	**0.035**
	≥ 477.40	15	11		21	13	
Total protein (g/L)	< 64.35	3	9	0.117	4	8	0.165
	≥ 64.35	15	14		23	18	
	NA	1					
Albumin (g/L)	< 39.45	4	12	0.089	6	14	**0.018**
	≥ 39.45	14	11		21	12	
	NA	1					
Prealbumin (g/L)	< 67.60	3	7	0.440^a^	9	12	0.291
	≥ 67.60	10	10		19	14	
	NA	12					
ALT (U/L)	< 12.00	6	4	0.468^a^	7	5	0.560
	≥ 12.00	13	19		20	21	
AST (U/L)	< 20.00	17	14	0.075^a^	24	17	**0.041**
	≥ 20.00	2	9		3	9	
Cystatin C (mg/L)	< 0.97	8	5	0.217	19	4	0.424
	≥ 0.97	11	16		22	8	
	NA	2					
Creatinine (umol/L)	< 71.00	14	11	0.089	21	14	0.066
	≥ 71.00	5	12		6	12	
Urine (mmol/L)	< 5.42	8	14	0.226	11	16	0.130
	≥ 5.42	11	9		16	10	
FDP (mg/L)	< 0.87	4	1	0.136^a^	10	9	0.422
	≥ 0.87	11	21		14	20	
	NA	5					
D-dimer (mg/L)	< 0.24	8	3	**0.012**^a^	**15**	**3**	**0.001**
	≥ 0.24	7	20		12	23	
	NA	4					
Fibrinogen (g/L)	< 2.60	1	5	0.216^a^	5	6	0.683
	≥ 2.60	16	18		22	20	
	NA	2					
TT (s)	< 16.85	6	15	0.061	16	16	0.610
	≥ 16.85	11	8		9	12	
	NA	2					
APTT (s)	< 35.20	12	21	0.113^a^	17	21	0.572
	≥ 35.20	5	2		8	7	
	NA	2					
PT (s)	< 12.65	16	17	0.205^a^	21	20	0.275
	≥ 12.65	1	6		4	8	
	NA	2					
CA199 (U/mL)	< 6.63	7	4	0.143^a^	12	9	0.340
	≥ 6.63	6	12		14	18	
	NA	13					
CEA (ng/mL)	< 2.71	8	4	**0.047**^a^	16	7	**0.018**
	≥ 2.71	5	12		11	19	
	NA	13					
SCCA (ng/mL)	< 2.51	12	11	0.183^a^	19	16	0.148
	≥ 2.51	1	5		6	12	
	NA	13					

^a^ Fisher exact test. ORR, objective response rate; WBC, white blood cell; NLR, neutrophil-to-lymphocytes ratio; PLR, platelet-to-lymphocyte ratio; SII, systemic immune-inflammation index; ALT, alanine aminotransferase; AST, aspartate aminotransferase; FDP, fibrin degradation product; TT, thrombin time; APTT, activated partial thromboplastin time; PT, prothrombin time; CA199, carbohydrate antigen199; CEA, carcinoembryonic antigen; SCCA, squamous cell carcinoma antigen.

Significant values are in [bold].

### Factors affecting the ORR of the patients with esophageal cancer after neoadjuvant chemotherapy combined with immunotherapy

To explore the factors that might affect the ORR after neoadjuvant therapy, we performed a univariate logistic regression analysis on all clinicopathological features and laboratory indicators of the patients. The result showed that neutrophil (< 5.22 × 10^9^/L), platelet (< 237.50 × 10^9^/L), PLR (< 166.50), SII (< 477.40), aspartate aminotransferase (AST) (≥ 20.00 U/L), D-dimer (≥ 0.24 mg/L) were associated with the ORR of the patients after neoadjuvant therapy (all *P* < 0.05) (Table 3). Thus, the above positive indicators were selected for further multivariate logistic regression analysis. Although the *P*-value of some indicators in the univariate regression analysis was greater than 0.05, in order not to omit the factors that might be related to ORR, we also enrolled the indicators with a *P*-value less than 0.1 [white blood cell (WBC), neutrophil-to-lymphocytes ratio (NLR), albumin, creatinine, fibrin degradation product (FDP), thrombin time (TT) and CEA)] into the subsequent multivariate logistic regression analysis (Table 3).

**Table 3:** Univariate logistic regression analysis of ORR in patients.

Term		OR	95%CI		*P*-value
			Lower	Upper	
Gender	Female versus Male	0.27	0.05	1.57	0.144
Age (Years)	≥ 52 versus < 52	5.87	0.60	57.79	0.130
BMI	≥ 22.57 versus < 22.57	1.87	0.54	6.46	0.323
Smoking history	Yes versus No	1.26	0.37	4.29	0.711
Drinking history	Yes versus No	0.60	0.15	2.40	0.472
Family history of cancer	Yes versus No	1.71	0.14	20.50	0.670
Tumor location	Median versus Upper	2.50	0.25	24.72	0.433
	Lower versus Upper	4.50	0.63	32.29	0.135
	Middle-lower versus Upper	3.75	0.54	26.04	0.181
Gross type	Other versus Medullary	2.23	0.64	7.74	0.207
Pathological type	Adenocarcinoma versus Squamous carcinoma	0.00	0.00		1.000
Grade	2/3 versus 1	1.34	0.30	6.02	0.703
cT stage	1/2 versus 3/4	0.78	0.12	5.26	0.802
cN stage	0/1 versus 2/3	0.72	0.19	2.74	0.626
cM stage	1 versus 0	3.79	0.39	37.20	0.253
cTNM stage	I/II versus versus III/IV	1.27	0.27	5.92	0.764
TP + PD-1 inhibitors	Sintilimab versus Camrelizumab	0.73	0.10	5.33	0.759
	Tireilizumab versus Camrelizumab	0.89	0.09	9.16	0.921
	Pembrolizumab versus versus Camrelizumab	0.83	0.09	7.68	0.872
Therapy cycle	3/4 versus 1/2	0.49	0.13	1.78	0.276
WBC (× 10^9^/L)	≥ 6.96 versus < 6.96	0.32	0.09	1.16	0.083
Lymphocyte (× 10^9^/L)	≥ 1.64 versus < 1.64	1.80	0.48	6.74	0.383
Neutrophil (× 10^9^/L)	≥ 5.22 versus < 5.22	0.17	0.04	0.76	**0.020**
Hemoglobin (g/L)	≥ 137.00 versus < 137.00	0.53	0.15	1.85	0.323
Platelet (× 10^9^/L)	≥ 237.50 versus < 237.50	0.26	0.07	0.92	**0.038**
NLR	≥ 2.42 versus < 2.42	0.31	0.09	1.10	0.071
PLR	≥ 166.50 versus < 166.50	0.26	0.07	0.94	**0.041**
SII	≥ 477.40 versus < 477.40	0.24	0.06	0.97	**0.044**
Total protein (g/L)	≥ 64.35 versus < 64.35	0.31	0.07	1.39	0.126
Albumin (g/L)	≥ 39.45 versus < 39.45	0.33	0.09	1.21	0.094
Prealbumin (g/L)	≥ 67.60 versus < 67.60	0.43	0.09	2.15	0.303
ALT (U/L)	≥ 12.00 versus < 12.00	2.19	0.51	9.33	0.288
AST (U/L)	≥ 20.00 versus < 20.00	5.46	1.01	29.54	**0.049**
Cystatin C (mg/L)	≥ 0.97 versus < 0.97	2.33	0.60	9.03	0.222
Creatinine (umol/L)	≥ 71.00 versus < 71.00	3.05	0.83	11.30	0.094
Urine (mmol/L)	≥ 5.42 versus < 5.42	0.47	0.14	1.61	0.228
FDP (mg/L)	≥ 0.87 versus < 0.87	7.64	0.76	76.90	0.084
D-dimer (mg/L)	≥ 0.24 versus < 0.24	7.62	1.57	37.05	**0.012**
Fibrinogen (g/L)	≥ 2.60 versus < 2.60	0.23	0.02	2.14	0.194
TT (s)	≥ 16.85 versus < 16.85	0.29	0.08	1.08	0.065
APTT (s)	≥ 35.20 versus < 35.20	0.23	0.04	1.36	0.105
PT (s)	≥ 12.65 versus < 12.65	5.65	0.61	52.22	0.127
CA199 (U/mL)	≥ 6.63 versus < 6.63	3.50	0.73	16.85	0.118
CEA (ng/mL)	≥ 2.71 versus < 2.71	4.80	0.98	23.54	0.053
SCCA (ng/mL)	≥ 2.51 versus < 2.51	5.45	0.55	54.28	0.148

ORR, objective response rate; OR, odds ratio; CI, confidence interval; BMI, body mass index; PD-1, programmed cell death protein-1; TP, platinum + taxol; WBC, white blood cell; NLR, neutrophil-to-lymphocytes ratio; PLR, platelet-to-lymphocyte ratio; SII, systemic immune-inflammation index; ALT, alanine aminotransferase; AST, aspartate aminotransferase; FDP, fibrin degradation product; TT, thrombin time; APTT, activated partial thromboplastin time; PT, prothrombin time; CA199, carbohydrate antigen199; CEA, carcinoembryonic antigen; SCCA, squamous cell carcinoma antigen.

Significant values are in [bold].

The result of the multivariate logistic regression analysis showed that AST, D-dimer and CEA were the independent predictors of the ORR after neoadjuvant TP regimen combined with PD-1 inhibitors (Table 4). All the three indicators were positively related to the ORR of the patients.

**Table 4:** Multivariate logistic regression analysis of ORR in patients.

Term		OR	95%CI		*P*-value
			Lower	Upper	
AST (U/L)	≥ 20.00 versus < 20.00	40.58	1.14	1439.47	**0.042**
D-dimer (mg/L)	≥ 0.24 versus < 0.24	99.85	1.28	7814.74	**0.038**
CEA (ng/mL)	≥ 2.71 versus < 2.71	48.04	1.35	1715.20	**0.034**

ORR, objective response rate; OR, odds ratio; CI, confidence interval; AST: CEA: carcinoembryonic antigen.

Significant values are in [bold].

### Establishment and validation of a nomogram for predicting the ORR of the patients after neoadjuvant therapy

**Figure 2:**
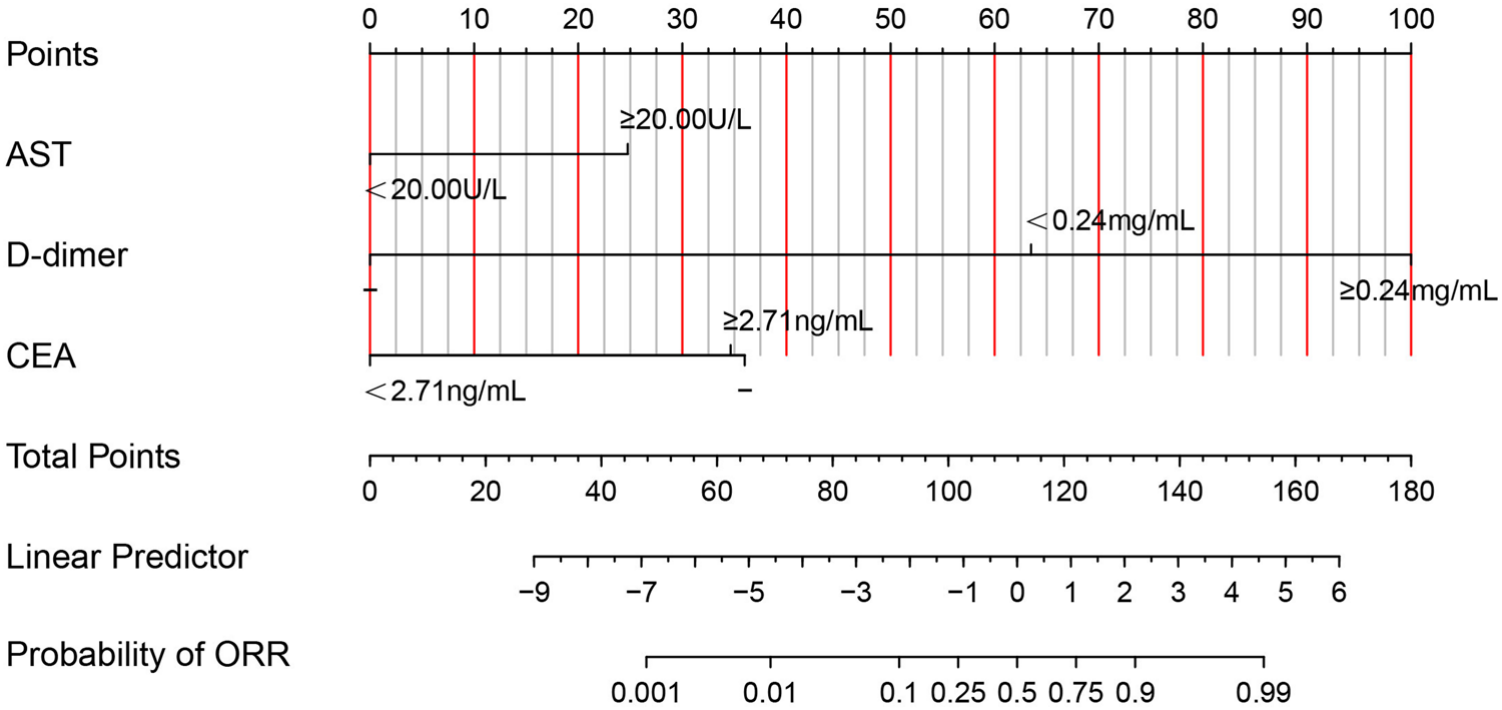
The nomogram for predicting the ORR of the patients after neoadjuvant TP regimen combined with PD-1 inhibitors. ORR, objective response rate; TP, taxol + platinum; PD-1, programmed cell death protein-1; AST, aspartate aminotransferase; CEA, carcinoembryonic antigen.

**Figure 3:**
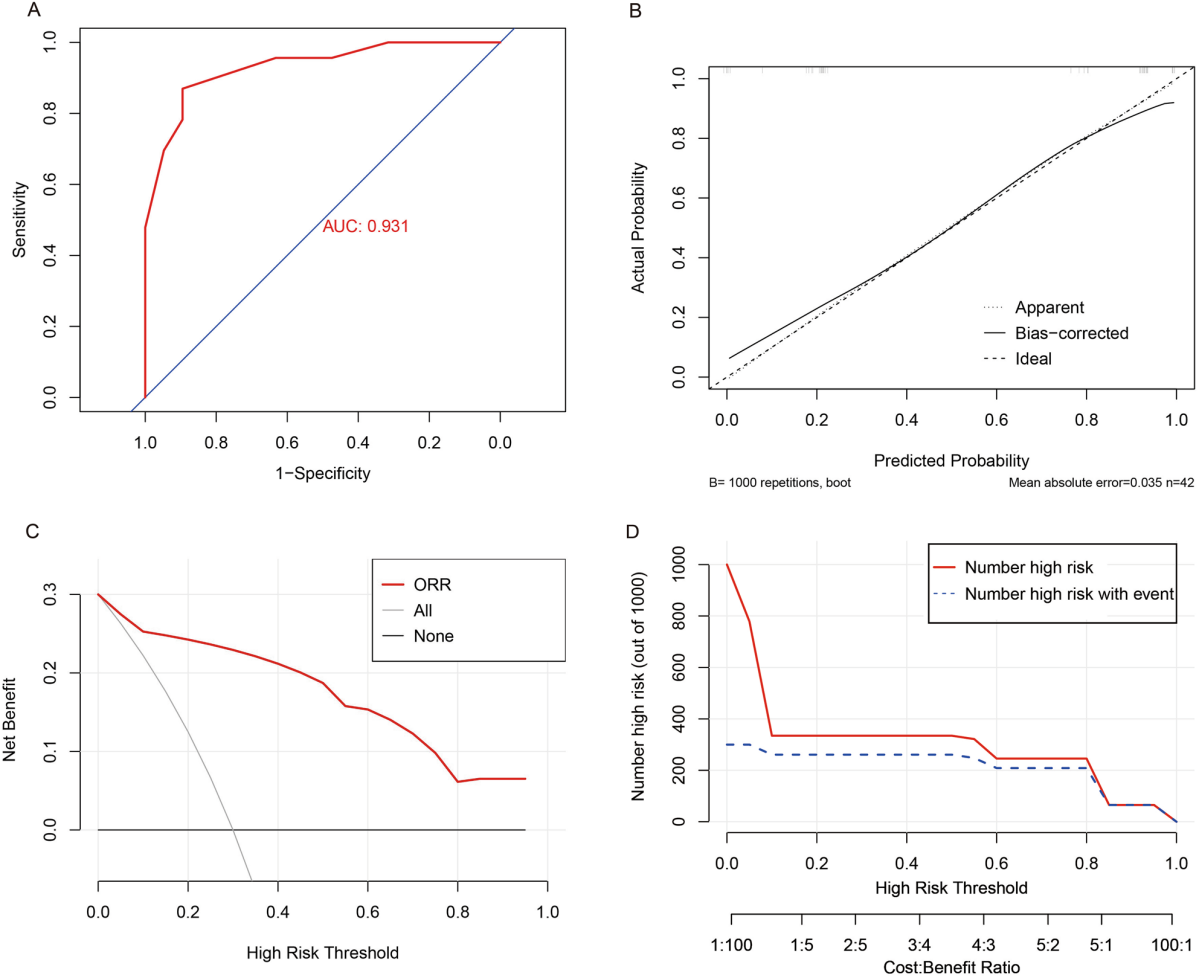
Internal validation of the nomogram for predicting the ORR after neoadjuvant therapy from training cohort. (**A**). Receiver operating characteristic curve. (**B**). Bootstrap validation curve. (**C**). Decision curve analysis. (**D**). Clinical impact curve. ORR, objective response rate.

According to the result of the multivariate logistic regression analysis, a nomogram based on AST, D-dimer and CEA was established to predict the ORR of the esophageal cancer patients who received neoadjuvant TP regimen combined with PD-1 inhibitors (Fig. 2). The C-index of the nomogram was 0.93 (95% CI 0.86–1.00). ROC curve of the nomogram showed that the value of area under curve (AUC) was 0.931 (Fig. 3A). Besides, the nomogram was validated by calibration curve (Fig. 3B) and the mean absolute error was 0.035. In addition, the DCA curve (Fig. 3C) and CIC curve (Fig. 3D) showed that the ability of the nomogram prediction model to predict pCR after NAC was pretty good.

**Figure 4:**
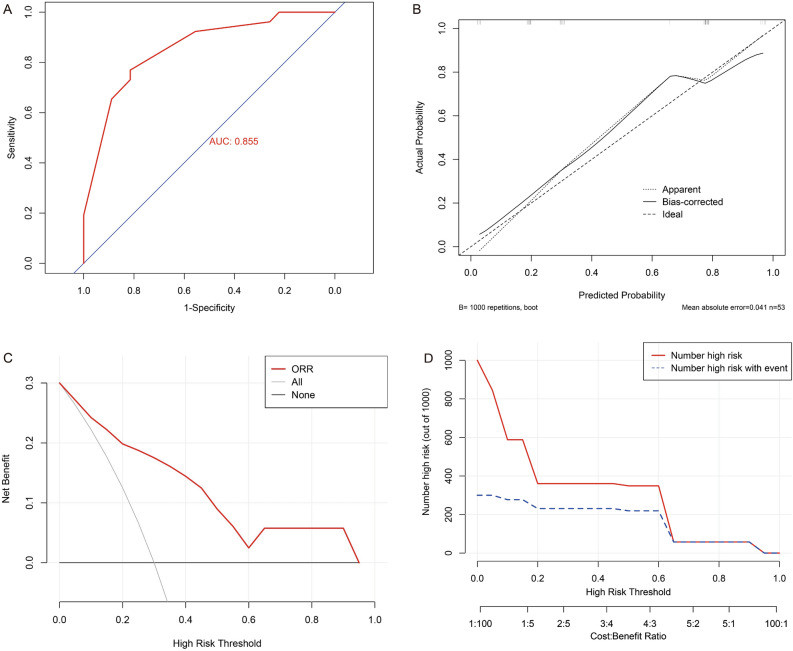
External verification of the nomogram for predicting the ORR after neoadjuvant therapy from validation cohort. (**A**). Receiver operating characteristic curve. (**B**). Bootstrap validation curve. (**C**). Decision curve analysis. (**D**). Clinical impact curve. ORR, objective response rate.

We also performed external validation of the nomogram by using validation cohort from Shaanxi Provincial Cancer Hospital Affiliated to Medical College of Xi'an Jiaotong University. The C-index of the validation cohort was 0.86 (95%CI: 0.76–0.95), while the AUC of ROC curve was 0.855 (Fig. 4A). The validation curve (Fig. 4B), DCA (Fig. 4C) and CIC (Fig. 4D) of the validation cohort also showed a good predictive ability of the nomogram.

## Discussion

This study evaluated the clinicopathological features and the laboratory hematological indexes that might affect the ORR of esophageal cancer patients after neoadjuvant therapy. Based on the positive factors, a nomogram for predicting ORR was established and verified.

The application of neoadjuvant therapy for esophageal cancer has greatly improved the prognosis of the patients^3,17^. The addition of immunotherapy to neoadjuvant therapy significantly prolonged the 5-year survival of the patients^18,19^. For locally advanced esophageal cancer, a number of prospective single-arm studies have confirmed the effectiveness and safety of neoadjuvant chemotherapy combined with immunotherapy^20–22^. After neoadjuvant therapy, these patients achieved high R0 resection rate, pCR rate and ORR. The subsequent problem is how to use accurate and simple methods to predict the efficacy of neoadjuvant therapy. Recently, some studies have reported predictive models that can predict the tumor regression grade (TRG) and pCR after neoadjuvant immunotherapy^13,14^. However, there is no related research on predicting ORR.

Previous studies have confirmed that inflammation and nutrition indexes before treatment were associated with the prognosis of the patients^23–25^. According to these indexes, the pathological response of the patients after neoadjuvant therapy can be effectively predicted^23–25^. For example, a recent study showed that the difference in albumin levels before and after neoadjuvant immunotherapy and WBC count before neoadjuvant therapy were significantly correlated with the TRG in patients^13^. Besides, inflammatory indicators, including NLR, PLR, LMR and SII, could well predict pCR after neoadjuvant immunization and participated in the development of various cancers^23,26,27^. In our study, we found that there were significant differences in neutrophil, platelet, PLR and SII between ORR group and non-ORR group. Univariate logistic regression analysis also showed that neutrophil, platelet, PLR and SII were significantly correlated with ORR. These results suggested that the expression level of these indexes before neoadjuvant therapy might affect the ORR of patients. Thus, in clinical practice, we might evaluate the efficacy of neoadjuvant immunotherapy by using these indexes.

D-dimer is a degradation product of fibrin and an indicator of hypercoagulability and endogenous fibrinolysis. It was reported that the plasma concentration of D-dimer was related to neoadjuvant chemotherapy efficacy and the prognosis in cancer^28^. The increase of plasma D-dimer level is related to the progression, increased lymph node metastases and poor prognosis of esophageal cancer^29,30^. Besides, plasma D-dimer was regarded as an independent prognostic factor for resectable esophageal cancer patients. The 5-year cancer-specific survival of patients with D-dimer ≤ 5.0 μg/mL was significantly better than that of patients with D-dimer > 0.5 μg/mL (35.5% vs. 21.1%)^31^. However, a previous study pointed out that a low D-dimer level was significantly and independently associated with better overall survival in lung cancer^27^. Similarly, in this study, we observed that elevated D-dimer level was related to ORR, suggesting the role of D-dimer in improved outcome of esophageal cancer patients with neoadjuvant immunotherapy. Moreover, serum AST is a biomarker of systemic inflammation and immune activation, which can be used to evaluate liver function^32^. AST was also associated with worse overall survival and a higher 90-day mortality rate after surgery in cancer patients^33^. But in esophageal cancer, serum AST/aspartate aminotransferase (ALT) level is a significant predictor of overall survival (OS). The 5-year OS of patients with high AST/ALT levels was longer than that of patients with low AST/ALT levels^34^. Besides, in non-virus-related hepatocellular carcinoma, AST was enrolled for constructing a prognostic model to evaluate the OS of patients, and the model showed a good predictive ability^35^. In this study, elevated AST was confirmed to contribute the improved ORR after neoadjuvant immunotherapy, which was consistent with the previous study^34^. As an accurate biomarker for occult advanced disease, preoperative serum CEA level could be used to predict the resectability of patients with esophageal cancer^36^. It was suggested that CEA could be used to predict the sensitivity of esophageal cancer to chemoradiotherapy^37^. In the present study, we found that CEA, AST and D-dimer were independently related to the ORR after neoadjuvant immunotherapy, indicating that these laboratory indicators before neoadjuvant therapy played an important role in predicting ORR of patients with esophageal cancer. Clinicians could combine these indicators to estimate the efficacy of neoadjuvant immunotherapy in patients with esophageal cancer.

The nomogram is a simple and useful tool for predicting outcome^38^. The nomogram model established according to the results of regression analysis can well predict the pathological response of the patients after neoadjuvant therapy^13,14,39,40^. The prediction effect of these models is pretty good. Based on the results of multivariate regression analysis, we also established a nomogram that could predict ORR. The C-index was 0.93 (95% CI 0.86–1.00), and the AUC value of ROC for the nomogram was 0.931, indicating a high predictive ability. Also, the results of the calibration curve, DCA curve and CIC curve showed that the prediction of the nomogram was pretty good. In addition, the external validation of the nomogram by using validation cohort from Shaanxi Provincial Cancer Hospital Affiliated to Medical College of Xi'an Jiaotong University showed similar results. All the results indicated that our nomogram had a good ability to predict ORR after neoadjuvant immunotherapy.

However, there are still some limitations that cannot be ignored in this study. First, this study is a retrospective analysis, so it is difficult to obtain data such as genetic testing (including PD-1 expression levels) of patients. Therefore, we only analyzed the clinicopathological factors and common laboratory indicators. Second, the sample size of this study is small. Thus, in the next step, we plan to conduct a prospective study, include more indexes and enlarge the sample size through multi-center cooperation to improve the prediction model.

## Conclusion

This study analyzed the clinicopathological factors and laboratory indexes that might affect the ORR of the patients with locally advanced esophageal cancer after neoadjuvant chemotherapy combined with immunotherapy. Based on the results of logistic regression analysis, a nomogram model for predicting ORR was established, and the model had good predictive ability. Our study provides a simple and feasible predictive model for ORR in patients with resectable locally advanced esophageal cancer after neoadjuvant TP regimen combined PD-1 inhibitors, which might be used in clinical practice for clinicians.

## Data Availability

The datasets generated during and/or analyzed during the current study are available from the corresponding author on reasonable request.
